# Approximation Set of the Interval Set in Pawlak's Space

**DOI:** 10.1155/2014/317387

**Published:** 2014-08-11

**Authors:** Qinghua Zhang, Jin Wang, Guoyin Wang, Feng Hu

**Affiliations:** ^1^The Chongqing Key Laboratory of Computational Intelligence, Chongqing University of Posts and Telecommunications, Chongqing 400065, China; ^2^School of Science, Chongqing University of Posts and Telecommunications, Chongqing 400065, China

## Abstract

The interval set is a special set, which describes uncertainty of an uncertain concept or set *Z* with its two crisp boundaries named upper-bound set and lower-bound set. In this paper, the concept of similarity degree between two interval sets is defined at first, and then the similarity degrees between an interval set and its two approximations (i.e., upper approximation set $\bar{R}$(*Z*) and lower approximation set $\underline{R}$(*Z*)) are presented, respectively. The disadvantages of using upper-approximation set $\bar{R}$(*Z*) or lower-approximation set $\underline{R}$(*Z*) as approximation sets of the uncertain set (uncertain concept) *Z* are analyzed, and a new method for looking for a better approximation set of the interval set *Z* is proposed. The conclusion that the approximation set *R*_0.5_(*Z*) is an optimal approximation set of interval set *Z* is drawn and proved successfully. The change rules of *R*_0.5_(*Z*) with different binary relations are analyzed in detail. Finally, a kind of crisp approximation set of the interval set *Z* is constructed. We hope this research work will promote the development of both the interval set model and granular computing theory.

## 1. Introduction

Since the twenty-first century, researchers have done more and more research on uncertain problems [1]. It is an important research topic on how to effectively deal with uncertain data and how to acquire more knowledge and rules from the big data. At the same time, many methods for acquiring uncertain knowledge from uncertain information systems appeared gradually. In 1965, fuzzy sets theory was proposed by Zadeh [2]. In 1982, rough sets theory was proposed by Pawlak [3]. In 1990, quotient space theory was presented by L. Zhang and B. Zhang [4]. In 1993, interval sets and interval sets algebra were presented by Yao [5, 6].

Rough set theory is a mathematical tool to handle the uncertain information, which is imprecise, inconsistent, or incomplete. The basic thought of rough set is to obtain concepts and rules through classification of relational database and discover knowledge by the classification induced by equivalence relations; then approximation sets of the target concept are obtained with many equivalence classes. Rough set is a useful tool to handle uncertain problems, as well as fuzzy set theory, probability theory, and evidence theory. Because rough set theory has novel ideas and its calculation is easy and simple, it has been an important technology in intelligent information processing [7–9]. The key issue of rough set is building a knowledge space which is a partition of the domain *U* and is induced by an equivalence relation. In the knowledge space, two certain sets named upper approximation set and lower approximation set are used to describe the target concept *X* as its two boundaries. If knowledge granularity in knowledge space is coarser, then the border region of described target concept is wider and approximate accuracy is relatively lower. On the contrary, if knowledge granularity in knowledge space is finer, then the border region is narrower and approximate accuracy is relatively higher.

The interval set theory is an effective method for describing ambiguous information [10–12] and can be used in uncertain reasoning as well as the rough set [13–15]. The interval set not only can be used to describe the partially known concept, but also can be used to study the approximation set of the uncertain target concept. So, the interval set is a more general model for processing the uncertain information [16]. The interval set is described by two sets named upper bound and lower bound [17]. The elements in lower bound certainly belong to target concept, and the elements in upper bound probably belong to target concept. When the boundary region has no element, the interval set degenerates into a usual set [5], while, in a certain knowledge granularity space, target concept may be uncertain. To solve this problem, in this paper, the approximate representation of interval set is discussed in detail in Pawlak's approximation space. And then, the upper approximation set of interval set and lower approximation set of interval set are defined, respectively. The change rules of the approximation set of interval set with the different knowledge granularity in Pawlak's approximation space are analyzed.

In this paper, an approximation set of the target concept *Z* is built in a certain knowledge space induced by many conditional attributes, and we find that this approximation set may have better similarity degree with the target concept *Z* than that of $\underline{R}(Z)$ or $\bar{R}(Z)$. Therefore, an interval set is translated into a fuzzy set at first in this paper. And then, according to the different membership degrees of different elements in boundary region, an approximation set of interval set *Z* is obtained by cut-set with some threshold. And then, the decision-making rules can be obtained through the approximation set instead of *Z* in current knowledge granularity space. In addition, the change rules of similarity between a target concept *Z* and its approximation sets are analyzed in detail.

The method used is getting the approximation of interval sets with a special approximation degree. With this method, we can use certain sets to describe an interval set in Pawlak's space. Our motivation is to get a mathematical theory model, which can be helpful to promote interval sets development in knowledge acquisition.

The rest of this paper is organized as follows. In Section 2, the related basic concepts and preliminary knowledge are reviewed. In Section 3, the concept of similarity degree between two interval sets is defined. The approximation set of interval set and 0.5-approximation set are proposed in Section 4. The change rules of similarity degree between the approximation sets and the target concept *Z* with the different knowledge granularity spaces are discussed in Section 5. This paper is concluded in Section 6.

## 2. Preliminaries

In order to introduce the approximation set of interval set more easily, many basic concepts will be reviewed at first.

**Definition 1 (interval set [17]).**

An interval set is a new collection, and it is described by two sets named upper bound and lower bound. The interval set can be defined as follows. Let *U* be a finite set which is called universal set, and then let 2^*U*^ be the power set of  *U* and let interval set *Z* be a subset of 2^*U*^. In mathematical form, interval set *Z* is defined as *Z* = [*Z*_*l*_, *Z*_*u*_] = {*Z* ∈ 2^*U*^∣*Z*_*l*_⊆*Z*⊆*Z*_*u*_}. If *Z*_*l*_ = *Z*_*u*_, *Z* is a usual classical set.

In order to better explain the interval set, there is an example [17, 18] as follows. Let *U* be all papers submitted to a conference. After being reviewed, there are 3 kinds of results. The first kind of results is the set of papers certainly accepted and represented by *Z*_*l*_. The second kind of results is the set of papers that need to be further reviewed and represented by *Z*_*u*_ − *Z*_*l*_. The last kind of results is the set of papers rejected and represented by *U* − *Z*_*u*_. Although every paper just can be rejected or accepted, no one knows the final result before further evaluation. Through reviewing, the set of papers accepted by the conference is described as [*Z*_*l*_, *Z*_*u*_].

**Definition 2 (indiscernibility relation [4, 19]).**

For any attribute set *R*⊆*A*, let us define one unclear binary relationship IND(*R*) = {(*x*, *y*)∣(*x*, *y*) ∈ *U* ^2^,  ∀*b* ∈ *R* → *b*(*x*) = *b*(*y*)}.

**Definition 3 (information table of knowledge expression system [4, 20]).**

A knowledge expression system can be described as *S* = 〈*U*, *A*, *V*, *f*〉. *U* is the domain, and *A* = *C* ∪ *D* is the set of all attributes. Subset *C* is a set of conditional attributes, and *D* is a set of decision-making attributes. *V* = ∪_*r*∈*A*_ *V*_*r*_ is the set of attribute values. *V*_*r*_ describes the range of attribute values *r* where *r* ∈ *A*. *f* : *U* × *A* → *V* is an information function which describes attribute values of object *x* in *U*.

**Definition 4 (upper approximation set and lower approximation set of rough set [3]).**

A knowledge-expression system is described as *S* = 〈*U*, *A*, *V*, *f*〉. For any *X*⊆*U* and *R*⊆*A*, upper approximation set $\bar{R}(X)$ and lower approximation set $\underline{R}(X)$ of rough set *X* on *R* are defined as follows:

(1) $$\begin{array}{c} \bar{R}\left(X\right)=\cup \left\{Y_{i}\;|\;Y_{i}\in \frac{U}{\text{IND}\left(R\right)}\wedge Y_{i}\cap X\neq \phi \right\},\\ \underline{R}\left(X\right)=\cup \left\{Y_{i}\;|\;Y_{i}\in \frac{U}{\text{IND}\left(R\right)}\wedge Y_{i}\subseteq X\right\}, \end{array}$$

where *U*/IND(*R*) = {*X*∣(*X*⊆*U*∧∀_*x*∈*X*,*y*∈*X*,__*b*∈*R*_(*b*(*x*) = *b*(*y*)))} is the classification of equivalence relation *R* on *U*. Upper approximation set and lower approximation set of rough set *X* on *R* can be defined in another form as follows:

(2) $$\begin{array}{c} \underline{R}\left(X\right)=\left\{x\;|\;x\in U\wedge {\left[x\right]}_{R}\subseteq X\right\},\\ \bar{R}\left(X\right)=\left\{x\;|\;x\in U\wedge {\left[x\right]}_{R}\cap X\neq \phi \right\}, \end{array}$$

where [*x*]_*R*_ ∈ *U*/IND(*R*) and [*x*]_*R*_ is an equivalence class of *x* on relation *R*. $\underline{R}(X)$ is a set of objects which certainly belong to *U* according to knowledge *R*; $\bar{R}(X)$ is a set of objects which possibly belong to *U* according to knowledge *R*. Let ${\text{BN}}_{R}(X)=\bar{R}(X)-\underline{R}(X)$ be called* boundary region* of target concept *X* on relation *R*. Let $\text{PO}{\text{S}}_{R}(X)=\underline{R}(X)$ be called* positive region* of target concept *X* on relation *R*. Let $\text{NE}{\text{G}}_{R}(X)=U-\bar{R}(X)$ be called* negative region* of target concept *X* on relation *R*. BN_*R*_(*X*) is a set of objects which just possibly belong to target concept *X*.

**Definition 5 (similarity degree between two sets [20]).**

Let *A* and *B* be two subsets of domain *U*, which means *A*⊆*U*,  *B*⊆*U*. Defining a mapping *S* : *U* × *U* → [0,1], that is, (*A*, *B*) → *S*(*A*, *B*), *S*(*A*, *B*) is the similarity degree between *A* and *B*, if *S*(*A*, *B*) satisfies the following conditions.

1. For any *A*, *B*⊆*U*,  0 ⩽ *S*(*A*, *B*) ⩽ 1 (boundedness).
2. For any *A*, *B*⊆*U*,  *S*(*A*, *B*) = *S*(*B*, *A*) (symmetry).
3. For any *A*, *B*⊆*U*,  *S*(*A*, *A*) = 1;  *S*(*A*, *B*) = 0 if and only if *A*∩*B* = *ϕ*.

Any formula satisfying (1), (2), and (3) is a similarity degree formula between two sets. Zhang et al. [20] gave out a similarity degree formula

(3) $$\begin{array}{c} S\left(A,B\right)=\frac{\left|A\cap B\right|}{\left|A\cup B\right|}, \end{array}$$

where |·| represents the number of elements in finite subset. Obviously, this formula satisfies (1), (2), and (3).

**Definition 6 (similarity degree between two interval sets).**

Let *Z* = [*Z*_*l*_, *Z*_*u*_] = {*Z* ∈ 2^*U*^∣*Z*_*l*_⊆*Z*⊆*Z*_*u*_} be an interval set and let *N* = [*N*_*l*_, *N*_*u*_] = {*N* ∈ 2^*U*^∣*N*_*l*_⊆*N*⊆*N*_*u*_} be also an interval set. Similarity degree between two interval sets can be defined as follows:

(4) $$\begin{array}{c} S\left(Z,N\right)=\frac{\left|Z_{l}\cap N_{l}\right|}{2\left|Z_{l}\cup N_{l}\right|}+\frac{\left|Z_{u}\cap N_{u}\right|}{2\left|Z_{u}\cup N_{u}\right|}. \end{array}$$

*S*(*Z*, *N*) accords with Definition 5.

**Definition 7 (upper approximation set and lower approximation set of an interval set).**

Let *Z* = [*Z*_*l*_, *Z*_*u*_] = {*Z* ∈ 2^*U*^∣*Z*_*l*_⊆*Z*⊆*Z*_*u*_} be an interval set. Let *R* be an equivalence relation on domain *U*. Upper approximation set of this interval set *Z* is defined as $\bar{R}\left(Z\right)=[\bar{R}\left(Z_{l}\right),\bar{R}\left(Z_{u}\right)]$. Lower approximation set of this interval set *Z* is defined as $\underline{R}\left(Z\right)=[\underline{R}\left(Z_{l}\right),\underline{R}\left(Z_{u}\right)]$.

**Figure 1:**
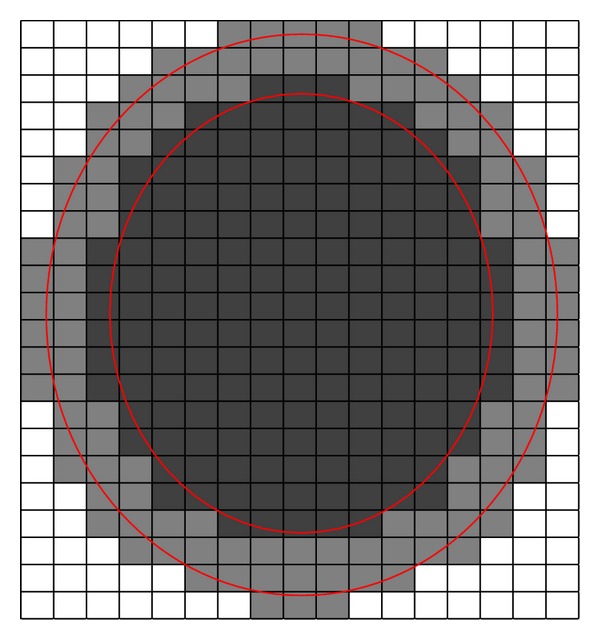
Upper approximation set of an interval set.

**Figure 2:**
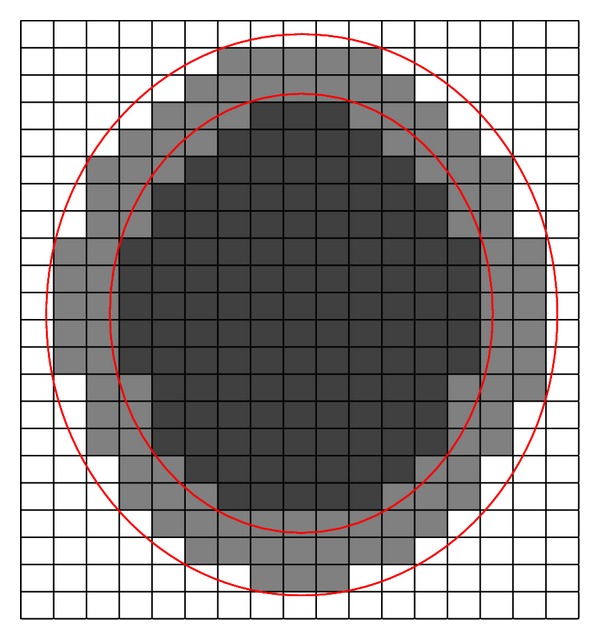
Lower approximation set of an interval set.

Figures 1 and 2 are probably helpful to understand Definition 7. In Figure 1, the outer circle standing for a set *Z*_*u*_ and inner circle standing for a set *Z*_*l*_ represent an interval set *Z*, and each block represents an equivalence class. The black region represents $\bar{R}\left(Z_{l}\right)$, and the whole colored region (black and gray region) represents $\bar{R}\left(Z_{u}\right)$. In Figure 2, the outer circle standing for a set *Z*_*u*_ and inner circle standing for a set *Z*_*l*_ represent an interval set *Z*, and each block represents an equivalence class. The black region represents $\underline{R}\left(Z_{l}\right)$, and the whole colored region (black and gray region) represents $\underline{R}\left(Z_{u}\right)$.

## 3. Approximation Set *R*_*λ*_(*Z*) of an Interval Set *Z*

If $\;\bar{R}\left(Z\right)$ stands for the upper approximation set of the interval set *Z*, then the similarity degree between *Z* and $\bar{R}\left(Z\right)$ can be defined as follows:

(5) S(Z,R¯(Z))=S(Zl,R¯(Zl))2+S(Zu,R¯(Zu))2=|Zl∩R¯(Zl)|2|Zl∪R¯(Zl)|+|Zu∩R¯(Zu)|2|Zu∪R¯(Zu)|.

If $\underline{R}\left(Z\right)$ stands for the lower approximation set of the interval set *Z*, then the similarity degree between *Z* and $\underline{R}\left(Z\right)$ is defined as follows:

(6) S(Z,R_(Z))=S(Zl,R_(Zl))2+S(Zu,R_(Zu))2=|Zl∩R_(Zl)|2|Zl∪R_(Zl)|+|Zu∩R_(Zu)|2|Zu∪R_(Zu)|.

If the knowledge space keeps unchanged, is there a better approximation set of the target concept *Z*? In this paper, the better approximation sets of target concept will be proposed. Let *U* be a nonempty set of objects. Let *Z*⊆*U*, *x* ∈ *Z*, and the membership degree of *x* belonging to set *Z* is defined as

(7) $$\begin{array}{c} \mu_{Z}^{R}\left(x\right)=\frac{\left|Z\cap {\left[x\right]}_{R}\right|}{\left|{\left[x\right]}_{R}\right|}. \end{array}$$

Obviously, 0 ⩽ *μ*_*Z*_^*R*^(*x*) ⩽ 1.

**Definition 8 (*λ*-approximation set of set *Z* [20]).**

Let *U* be a nonempty set of objects, and let knowledge space be *U*/IND(*R*). Let *Z*⊆*U*,  *x* ∈ *Z*, and the membership degree belonging to set *Z* is

(8) $$\begin{array}{c} \mu_{Z}^{R}\left(x\right)=\frac{\left|Z\cap {\left[x\right]}_{R}\right|}{\left|{\left[x\right]}_{R}\right|}. \end{array}$$

If  *R*_*λ*_(*Z*) = {*x* ∈ *Z*∣*μ*_*Z*_^*R*^(*x*)⩾*λ*,  1⩾*λ* > 0}, then *R*_*λ*_(*Z*) is called *λ*-approximation set of set *Z*.

**Definition 9 (*λ*-approximation set of set *Z*).**

Let *Z* = [*Z*_*l*_, *Z*_*u*_] = {*Z* ∈ 2^*U*^∣*Z*_*l*_⊆*Z*⊆*Z*_*u*_} and *R*_*λ*_(*Z*) = [*R*_*λ*_(*Z*_*l*_), *R*_*λ*_(*Z*_*u*_)]; then *R*_*λ*_(*Z*) is called *λ*-approximation set of the interval set *Z*.

**Figure 3:**
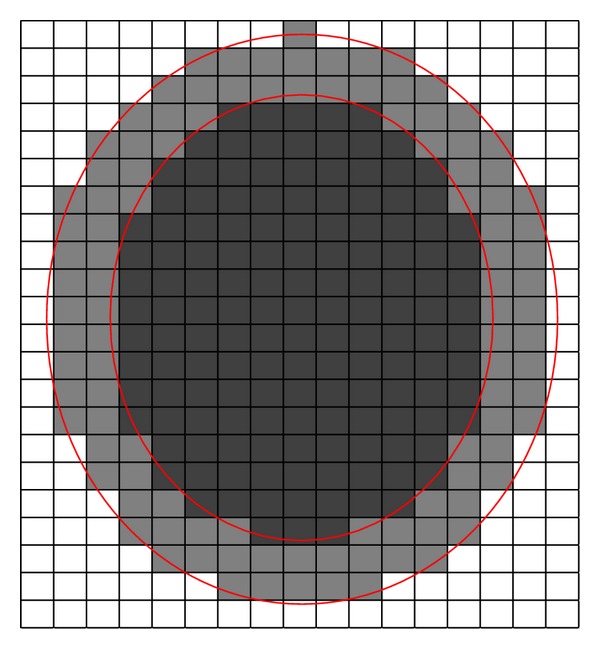
0.5-approximation set of an interval set.

Figure 3 is probably helpful to understand Definition 9. In Figure 3, the outer circle standing for a set *Z*_*u*_ and inner circle standing for a set *Z*_*l*_ represent an interval set *Z*, and each block represents an equivalence class. The black region represents *R*_0.5_(*Z*_*l*_), and the whole colored region (black and gray region) represents *R*_0.5_(*Z*_*u*_).

## 4. Approximation Set *R*_0.5_(*Z*) of an Interval Set *Z*

**Lemma 10 (see [20]).**

Let *a*, *b*, *c*, and *d* be all real numbers. If 0 < *a* < *b*,  0 < *c* < *d*, then *a*/*b* < (*a* + *d*)/(*b* + *c*).

**Lemma 11 (see [20]).**

Let *a*, *b*, *c*, and *d* be all real numbers. In the numbers, 0 < *a* < *b*,  0 < *c* < *d*. If *a*/*b*⩾*c*/*d*, then *a*/*b* ⩽ (*a* − *c*)/(*b* − *d*). If  *a*/*b* ⩽ *c*/*d*, then *a*/*b*⩾(*a* − *c*)/(*b* − *d*).

In order to better understand the similarity degree between *R*_0.5_(*Z*) and *Z*, Theorems 12 and 13 are presented as follows.

**Theorem 12 .**

Let *U* be a finite domain, let *Z* be an interval set on *U*, and let *R* be an equivalence relation on *U*. Then, $S(Z,R_{0.5}(Z))⩾S(Z,\underline{R}(Z))$.

For example, let *U*/*R* = {{*x*_1_, *x*_2_}, {*x*_3_, *x*_4_}, {*x*_5_, *x*_6_}}, *Z*_*u*_ = {*x*_1_, *x*_2_, *x*_3_, *x*_4_, *x*_5_}, *Z*_*l*_ = {*x*_2_, *x*_3_, *x*_4_}. Then, $\underline{R}\left(Z_{u}\right)=\left\{x_{1},x_{2},x_{3},x_{4}\right\}$, $\bar{R}\left(Z_{u}\right)=\left\{x_{1},x_{2},x_{3},x_{4},x_{5},x_{6}\right\}$, *R*_0.5_(*Z*_*u*_) = {*x*_1_, *x*_2_, *x*_3_, *x*_4_, *x*_5_, *x*_6_}, $\underline{R}\left(Z_{l}\right)=\left\{x_{3},x_{4}\right\}$, $\bar{R}\left(Z_{l}\right)=\left\{x_{1},x_{2},x_{3},x_{4}\right\}$, *R*_0.5_(*Z*_*l*_) = {*x*_1_, *x*_2_, *x*_3_, *x*_4_}.

And then we can have $S\left(Z,\underline{R}\left(Z\right)\right)=\left(2/(2\times 3)\right)+\left(4/(2\times 5)\right)=11/15$, $S\left(Z,\bar{R}\left(Z\right)\right)=\left(5/(2\times 6)\right)+\left(3/(2\times 4)\right)=19/24$, *S*(*Z*, *R*_0.5_(*Z*)) = (1/2) + (3/(2 × 4)) = 7/8, $S\left(Z,R_{0.5}\left(Z\right)\right)⩾S\left(Z,\underline{R}\left(Z\right)\right)$.

**Proof**

According to Definition 6,

(9) $$\begin{array}{c} S\left(Z,R_{0.5}\left(Z\right)\right)=\frac{\left|Z_{l}\cap R_{0.5}\left(Z_{l}\right)\right|}{2\left|Z_{l}\cup R_{0.5}\left(Z_{l}\right)\right|}+\frac{\left|Z_{u}\cap R_{0.5}\left(Z_{u}\right)\right|}{2\left|Z_{u}\cup R_{0.5}\left(Z_{u}\right)\right|},\\ S\left(Z,\underline{R}\left(Z\right)\right)=\frac{\left|Z_{l}\cap \underline{R}\left(Z_{l}\right)\right|}{2\left|Z_{l}\cup \underline{R}\left(Z_{l}\right)\right|}+\frac{\left|Z_{u}\cap \underline{R}\left(Z_{u}\right)\right|}{2\left|Z_{u}\cup \underline{R}\left(Z_{u}\right)\right|}. \end{array}$$

(1) There we first prove

(10) $$\begin{array}{c} \frac{\left|Z_{l}\cap R_{0.5}\left(Z_{l}\right)\right|}{2\left|Z_{l}\cup R_{0.5}\left(Z_{l}\right)\right|}⩾\frac{\left|Z_{l}\cap \underline{R}\left(Z_{l}\right)\right|}{2\left|Z_{l}\cup \underline{R}\left(Z_{l}\right)\right|}. \end{array}$$

For all *x* ∈ *R*_0.5_(*Z*_*l*_), we have *μ*_*Z*_*l*__^*R*^(*x*)⩾0.5. That is,

(11) $$\begin{array}{c} \mu_{Z_{l}}^{R}\left(x\right)=\frac{\left|{\left[x\right]}_{R}\cap Z_{l}\right|}{\left|{\left[x\right]}_{R}\right|}⩾0.5. \end{array}$$

Because *R* is an equivalence relation on *U*, the classifications induced by *R* can be denoted as [*x*_1_]_*R*_, [*x*_2_]_*R*_,…, [*x*_*n*_]_*R*_. Then, *R*_0.5_(*Z*_*l*_) = {*x*∣*μ*_*Z*_*l*__^*R*^(*x*)⩾0.5} = {*x*∣*μ*_*Z*_*l*__^*R*^(*x*) = 1}∪{*x*∣0.5 ⩽ *μ*_*X*_^*R*^(*x*) < 1}. Obviously, $\left\{x|\mu_{Z_{l}}^{R}(x)=1\}=\underline{R}(Z_{l}\right)$, and then let {*x*∣0.5 ⩽ *μ*_*Z*_*l*__^*R*^(*x*) < 1} = [*x*_*i*_1__]_*R*_ ∪ [*x*_*i*_2__]_*R*_ ∪ ⋯∪[*x*_*i*_*k*__]_*R*_. So, $\;Z_{l}\cap R_{0.5}(Z_{l})=Z_{l}\cap (\underline{R}(Z_{l})\cup {\left[x_{i_{1}}\right]}_{R}\cup {\left[x_{i_{2}}\right]}_{R}\cup \cdots \cup {\left[x_{i_{k}}\right]}_{R})$. Because the intersection sets between any two elements in $\underline{R}(Z_{l}),{\left[x_{i_{1}}\right]}_{R},{\left[x_{i_{2}}\right]}_{R},\ldots ,{\left[x_{i_{k}}\right]}_{R}$ are empty sets, we can get that

(12) |Zl∩R0.5(Zl)|=|Zl∩R_(Zl)|+|Zl∩[xi1]R|+|Zl∩[xi2]R|+⋯+|Zl∩[xik]R|=|R_(Zl)|+|Zl∩[xi1]R|+|Zl∩[xi2]R|+⋯+|Zl∩[xik]R|.

Because  *Z*_*l*_ ∪ *R*_0.5_(*Z*_*l*_) = *Z*_*l*_ ∪ ([*x*_*i*_1__]_*R*_ − *Z*_*l*_)∪([*x*_*i*_2__]_*R*_ − *Z*_*l*_)∪⋯∪([*x*_*i*_*k*__]_*R*_ − *Z*_*l*_) and the intersection set between any two elements in  *Z*_*l*_, ([*x*_*i*_1__]_*R*_ − *Z*_*l*_), ([*x*_*i*_2__]_*R*_ − *Z*_*l*_),…, ([*x*_*i*_*k*__]_*R*_ − *Z*_*l*_) is empty, we have that |*Z*_*l*_ ∪ *R*_0.5_(*Z*_*l*_)| = |*Z*_*l*_ | +|([*x*_*i*_1__]_*R*_ − *Z*_*l*_)|+|([*x*_*i*_2__]_*R*_ − *Z*_*l*_)|+⋯ + |([*x*_*i*_*k*__]_*R*_ − *Z*_*l*_)|. So,

(13) |Zl∩R0.5(Zl)||Zl∪R0.5(Zl)| =(|R_(Zl)|+|Zl∩[xi1]R|+|Zl∩[xi2]R|  +⋯+|Zl∩[xik]R|)  ×(|Zl|+|([xi1]R−Zl)|+|([xi2]R−Zl)|  +⋯+|([xik]R−Zl)|)−1.

Because

(14) μZlR(xi1)=|[xi1]R∩Zl||[xi1]R|=|[xi1]R∩Zl||[xi1]R∩Zl|+|[xi1]R−Zl|⩾0.5,

we have |[*x*_*i*_1__]_*R*_∩*Z*_*l*_ | ⩾|[*x*_*i*_1__]_*R*_ − *Z*_*l*_|. In the same way, according to |[*x*_*i*_2__]_*R*_∩*Z*_*l*_ | ⩾|[*x*_*i*_2__]_*R*_ − *Z*_*l*_ | ,…, |[*x*_*i*_*k*__]_*R*_∩*Z*_*l*_ | ⩾|[*x*_*i*_*k*__]_*R*_ − *Z*_*l*_| and Lemma 10, we can easily get

(15) |Zl∩R0.5(Zl)||Zl∪R0.5(Zl)| =(|R_(Zl)|+|Zl∩[xi1]R|+|Zl∩[xi2]R|  +⋯+|Zl∩[xik]R|)  ×(|Zl|+|([xi1]R−Zl)|+|([xi2]R−Zl)|  +⋯+|([xik]R−Zl)|)−1 ⩾|R_(Zl)||Zl|.

Therefore,

(16) $$\begin{array}{c} \frac{\left|Z_{l}\cap R_{0.5}\left(Z_{l}\right)\right|}{2\left|Z_{l}\cup R_{0.5}\left(Z_{l}\right)\right|}⩾\frac{\left|Z_{l}\cap \underline{R}\left(Z_{l}\right)\right|}{2\left|Z_{l}\cup \underline{R}\left(Z_{l}\right)\right|}. \end{array}$$

(2) In a similar way with (1), we can have the inequality

(17) $$\begin{array}{c} \frac{\left|Z_{u}\cap R_{0.5}\left(Z_{u}\right)\right|}{2\left|Z_{u}\cup R_{0.5}\left(Z_{u}\right)\right|}⩾\frac{\left|Z_{u}\cap \underline{R}\left(Z_{u}\right)\right|}{2\left|Z_{u}\cup \underline{R}\left(Z_{u}\right)\right|}. \end{array}$$

From (1) and (2), we have $S(Z,R_{0.5}(Z))⩾S(Z,\underline{R}(Z))$. So, Theorem 12 has been proved completely.

Theorem 12 shows that the similarity degree between an interval set *Z* and its approximation set *R*_0.5_(*Z*) is better than the similarity degree between *Z* and its lower approximation set $\underline{R}(Z)$.

**Theorem 13 .**

Let *U* be a finite domain, let *Z* be an interval set on *U*, and let *R* be an equivalence relation on *U*. If

(18) $$\begin{array}{c} \frac{\left|Z_{l}\right|}{\left|\bar{R}\left(Z_{l}\right)\right|}>\frac{\left|Z_{l}-R_{0.5}\left(Z_{l}\right)\right|}{\left|\bar{R}\left(Z_{l}\right)-R_{0.5}\left(Z_{l}\right)-Z_{l}\right|},\\ \frac{\left|Z_{u}\right|}{\left|\bar{R}\left(Z_{u}\right)\right|}>\frac{\left|Z_{u}-R_{0.5}\left(Z_{u}\right)\right|}{\left|\bar{R}\left(Z_{u}\right)-R_{0.5}\left(Z_{u}\right)-Z_{u}\right|}, \end{array}$$

then $S(Z,R_{0.5}(Z))⩾S(Z,\bar{R}(Z))$.

For example, let *U*/*R* = {{*x*_1_}, {*x*_2_, *x*_3_, *x*_4_, *x*_5_, *x*_6_}, {*x*_7_, *x*_8_, *x*_9_, *x*_10_}},  *Z*_*u*_ = {*x*_1_, *x*_2_, *x*_7_}, *Z*_*l*_ = {*x*_1_, *x*_2_}. Then, $\bar{R}\left(Z_{u}\right)=\left\{x_{1},x_{2},x_{3},x_{4},x_{5},x_{6},x_{7},x_{8},x_{9},x_{10}\right\}$, $\underline{R}\left(Z_{u}\right)=\left\{x_{1}\right\}$, *R*_0.5_(*Z*_*u*_) = {*x*_1_}, $\underline{R}\left(Z_{l}\right)=\left\{x_{1}\right\}$, $\bar{R}\left(Z_{l}\right)=\left\{x_{1},x_{2},x_{3},x_{4},x_{5},x_{6}\right\}$, *R*_0.5_(*Z*_*l*_) = {*x*_1_},

(19) $$\begin{array}{c} \frac{\left|Z_{l}\right|}{\left|\bar{R}\left(Z_{l}\right)\right|}=\frac{2}{6}=\frac{1}{3}>\frac{\left|Z_{l}-R_{0.5}\left(Z_{l}\right)\right|}{\left|\bar{R}\left(Z_{l}\right)-Z_{l}-R_{0.5}\left(Z_{l}\right)\right|}=\frac{1}{4},\\ \frac{\left|Z_{u}\right|}{\left|\bar{R}\left(Z_{u}\right)\right|}=\frac{3}{10}>\frac{\left|Z_{u}-R_{0.5}\left(Z_{u}\right)\right|}{\left|\bar{R}\left(Z_{u}\right)-Z_{u}-R_{0.5}\left(Z_{u}\right)\right|}=\frac{2}{7}. \end{array}$$

And then we can have *S*(*Z*, *R*_0.5_(*Z*)) = (1/(2 × 2)) + (1/(2 × 3)) = 5/12,$\;S\left(Z,\bar{R}\left(Z\right)\right)=\left(1/(2\times 6)\right)+\left(3/(2\times 10)\right)=7/30$, $S\left(Z,R_{0.5}\left(Z\right)\right)⩾S\left(Z,\bar{R}\left(Z\right)\right)$.

**Proof**

According to Definition 6,

(20) $$\begin{array}{c} S\left(Z,R_{0.5}\left(Z\right)\right)=\frac{\left|Z_{l}\cap R_{0.5}\left(Z_{l}\right)\right|}{2\left|Z_{l}\cup R_{0.5}\left(Z_{l}\right)\right|}+\frac{\left|Z_{u}\cap R_{0.5}\left(Z_{u}\right)\right|}{2\left|Z_{u}\cup R_{0.5}\left(Z_{u}\right)\right|},\\ S\left(Z,\bar{R}\left(Z\right)\right)=\frac{\left|Z_{l}\cap \bar{R}\left(Z_{l}\right)\right|}{2\left|Z_{l}\cup \bar{R}\left(Z_{l}\right)\right|}+\frac{\left|Z_{u}\cap \bar{R}\left(Z_{u}\right)\right|}{2\left|Z_{u}\cup \bar{R}\left(Z_{u}\right)\right|}. \end{array}$$

(1) Let $\bar{R}(Z_{l})-R_{0.5}(Z_{l})={\left[x_{j_{1}}\right]}_{R}\cup {\left[x_{j_{2}}\right]}_{R}\cup \cdots \cup {\left[x_{j_{s}}\right]}_{R}$, and the intersection sets between any two elements in [*x*_*j*_1__]_*R*_, [*x*_*j*_2__]_*R*_,…, [*x*_*j*_*s*__]_*R*_ are empty sets. Because

(21) $$\begin{array}{c} 0<\mu_{Z_{l}}^{R}\left(x_{j_{1}}\right)=\frac{\left|{\left[x_{j_{1}}\right]}_{R}\cap Z_{l}\right|}{\left|{\left[x_{j_{1}}\right]}_{R}\right|}<0.5, \end{array}$$

it is obvious that [*x*_*j*_1__]_*R*_∩*Z*_*l*_ ≠ *ϕ*. In the same way, we have [*x*_*j*_2__]_*R*_∩*Z*_*l*_ ≠ *ϕ*,…, [*x*_*j*_*s*__]_*R*_∩*Z*_*l*_ ≠ *ϕ*. Then we have *Z*_*l*_∩*R*_0.5_(*Z*_*l*_) = *Z*_*l*_ − ([*x*_*j*_1__]_*R*_ ∩ *Z*_*l*_)−([*x*_*j*_2__]_*R*_ ∩ *Z*_*l*_)−⋯ − ([*x*_*j*_*s*__]_*R*_ ∩ *Z*_*l*_) = *Z*_*l*_ − (*Z*_*l*_ − *R*_0.5_(*Z*_*l*_)). Because the intersection sets between any two elements in [*x*_*j*_1__]_*R*_∩*Z*_*l*_, [*x*_*j*_2__]_*R*_∩*Z*_*l*_,…, [*x*_*j*_*s*__]_*R*_∩*Z*_*l*_ are empty sets, we have *Z*_*l*_∩*R*_0.5_(*Z*_*l*_) = *Z*_*l*_ − (*Z*_*l*_ − *R*_0.5_(*Z*_*l*_)), |*Z*_*l*_∩*R*_0.5_(*Z*_*l*_)| = |*Z*_*l*_ | −|*Z*_*l*_ − *R*_0.5_(*Z*_*l*_)|, and $Z_{l}\cup R_{0.5}(Z_{l})=\bar{R}(Z_{l})-(({\left[x_{j_{1}}\right]}_{R}-Z_{l})\cup ({\left[x_{j_{2}}\right]}_{R}-Z_{l})$ *∪*⋯∪([*x*_*j*_*s*__]_*R*_ − *Z*_*l*_)). Because the intersection sets between any two elements in ([*x*_*j*_1__]_*R*_ − *Z*_*l*_), ([*x*_*j*_2__]_*R*_ − *Z*_*l*_),…, ([*x*_*j*_*s*__]_*R*_ − *Z*_*l*_) are empty sets, $Z_{l}\cup R_{0.5}(Z_{l})=\bar{R}(Z_{l})-(\bar{R}(Z_{l})-R_{0.5}(Z_{l})-Z_{l})$ and $\left|Z_{l}\cup R_{0.5}(Z_{l})|=|\bar{R}(Z_{l})|-|(\bar{R}(Z_{l})-R_{0.5}(Z_{l})-Z_{l})\right|$ are held. So,

(22) $$\begin{array}{c} \frac{\left|Z_{l}\cap R_{0.5}\left(Z_{l}\right)\right|}{\left|Z_{l}\cup R_{0.5}\left(Z_{l}\right)\right|}=\frac{\left|Z_{l}\right|-\left|Z_{l}-R_{0.5}\left(Z_{l}\right)\right|}{\left|\bar{R}\left(Z_{l}\right)\right|-\left|\left(\bar{R}\left(Z_{l}\right)-R_{0.5}\left(Z_{l}\right)-Z_{l}\right)\right|}. \end{array}$$

For

(23) $$\begin{array}{c} \frac{\left|Z_{l}\right|}{\left|\bar{R}\left(Z_{l}\right)\right|}>\frac{\left|Z_{l}-R_{0.5}\left(Z_{l}\right)\right|}{\left|\bar{R}\left(Z_{l}\right)-R_{0.5}\left(Z_{l}\right)-Z_{l}\right|}, \end{array}$$

according to Lemma 11, we have

(24) $$\begin{array}{c} \frac{\left|Z_{l}\right|}{\left|\bar{R}\left(Z_{l}\right)\right|}⩽\frac{\left|Z_{l}\right|-\left|Z_{l}-R_{0.5}\left(Z_{l}\right)\right|}{\left|\bar{R}\left(Z_{l}\right)\right|-\left|\left(\bar{R}\left(Z_{l}\right)-R_{0.5}\left(Z_{l}\right)-Z_{l}\right)\right|}; \end{array}$$

that is to say,

(25) $$\begin{array}{c} \frac{\left|Z_{l}\cap R_{0.5}\left(Z_{l}\right)\right|}{\left|Z_{l}\cup R_{0.5}\left(Z_{l}\right)\right|}⩾\frac{\left|Z_{l}\cap \bar{R}\left(Z_{l}\right)\right|}{\left|Z_{l}\cup \bar{R}\left(Z_{l}\right)\right|}. \end{array}$$

Therefore, we have

(26) $$\begin{array}{c} \frac{\left|Z_{l}\cap R_{0.5}\left(Z_{l}\right)\right|}{2\left|Z_{l}\cup R_{0.5}\left(Z_{l}\right)\right|}⩾\frac{\left|Z_{l}\cap \bar{R}\left(Z_{l}\right)\right|}{2\left|Z_{l}\cup \bar{R}\left(Z_{l}\right)\right|}. \end{array}$$

(2) In a similar way with (1), we can easily obtain the conclusion that

(27) $$\begin{array}{c} \frac{\left|Z_{u}\cap R_{0.5}\left(Z_{u}\right)\right|}{2\left|Z_{u}\cup R_{0.5}\left(Z_{u}\right)\right|}⩾\frac{\left|Z_{u}\cap \bar{R}\left(Z_{u}\right)\right|}{2\left|Z_{u}\cup \bar{R}\left(Z_{u}\right)\right|} \end{array}$$

when

(28) $$\begin{array}{c} \frac{\left|Z_{u}\right|}{\left|\bar{R}\left(Z_{u}\right)\right|}>\frac{\left|Z_{u}-R_{0.5}\left(Z_{u}\right)\right|}{\left|\bar{R}\left(Z_{u}\right)-R_{0.5}\left(Z_{u}\right)-Z_{u}\right|}. \end{array}$$

According to (1) and (2), the inequality $S(Z,R_{0.5}(Z))⩾S(Z,\bar{R}(Z))$ is held. So, Theorem 13 has been proved successfully.

Theorem 13 shows that, under some conditions, the similarity degree between an interval set *Z* and its approximation set *R*_0.5_(*Z*) is better than the similarity degree between *Z* and its lower approximation set $\bar{R}(Z)$.

**Theorem 14 .**

Let *U* be a finite domain, *Z* an interval set on *U*, and *R* an equivalence relation on *U*. If 1⩾*λ* > 0.5, then $S(Z,R_{0.5}(Z))⩾S(Z,R_{\lambda }(Z))⩾S(Z,\underline{R}(Z))$.

For example, let *U*/*R* = {{*x*_1_}, {*x*_2_, *x*_3_}, {*x*_4_, *x*_5_, *x*_6_}},  *Z*_*u*_ = {*x*_1_, *x*_2_, *x*_3_, *x*_4_, *x*_5_}, *Z*_*l*_ = {*x*_1_, *x*_2_, *x*_3_}. Then, $\bar{R}\left(Z_{u}\right)=\left\{x_{1},x_{2},x_{3},x_{4},x_{5},x_{6}\right\}$, *R*_0.5_(*Z*_*u*_) = {*x*_1_, *x*_2_, *x*_3_, *x*_4_, *x*_5_, *x*_6_}, $\underline{R}\left(Z_{u}\right)=\left\{x_{1},x_{2},x_{3}\right\}$, *R*_0.75_(*Z*_*u*_) = {*x*_1_, *x*_2_, *x*_3_},$\;\;\underline{R}\left(Z_{l}\right)=\left\{x_{1},x_{2},x_{3}\right\}$, $\bar{R}\left(Z_{l}\right)=\left\{x_{1},x_{2},x_{3}\right\}$, and *R*_0.5_(*Z*_*l*_) = {*x*_1_, *x*_2_, *x*_3_}, *R*_0.75_(*Z*_*l*_) = {*x*_1_, *x*_2_, *x*_3_}.

And then we can have *S*(*Z*, *R*_0.5_(*Z*)) = (3/(2 × 3)) + (5/(2 × 6)) = 11/12, *S*(*Z*, *R*_0.75_(*Z*)) = (3/(2 × 3)) + (3/(2 × 5)) = 4/5, $S\left(Z,\underline{R}\left(Z\right)\right)=\left(3/(2\times 3)\right)+\left(3/(2\times 6)\right)=3/4$, $S\left(Z,\underline{R}\left(Z\right)\right)<S\left(Z,R_{0.75}\left(Z\right)\right)<S\left(Z,R_{0.5}\left(Z\right)\right)$. This example is in accordance with the theorem.

**Proof**

(1) For all *x* ∈ *R*_0.5_(*Z*_*l*_), then *μ*_*Z*_*l*__^*R*^(*x*)⩾0.5, which means

(29) $$\begin{array}{c} \mu_{Z_{l}}^{R}\left(x\right)=\frac{\left|{\left[x\right]}_{R}\cap Z_{l}\right|}{\left|{\left[x\right]}_{R}\right|}⩾0.5. \end{array}$$

Because *R*_0.5_(*Z*_*l*_) = {*x*∣*μ*_*Z*_*l*__^*R*^(*x*)⩾0.5} = {*x*∣*μ*_*Z*_*l*__^*R*^(*x*) = 1} ∪ {*x*∣0.5 ⩽ *μ*_*Z*_*l*__^*R*^(*x*) < 1}, we can easily get $\left\{x|\mu_{Z_{l}}^{R}(x)=1\}=\underline{R}(Z_{l}\right)$. Let {*x*∣0.5 ⩽ *μ*_*Z*_*l*__^*R*^(*x*) < 1} = [*x*_*i*_1__]_*R*_ ∪ [*x*_*i*_2__]_*R*_ ∪ ⋯∪[*x*_*i*_*k*__]_*R*_ and *R*_*λ*_(*Z*_*l*_) = {*x*∣*μ*_*Z*_*l*__^*R*^(*x*)⩾*λ* > 0.5} = {*x*∣*μ*_*Z*_*l*__^*R*^(*x*) = 1}∪{*x*∣0.5 < *λ* ⩽ *μ*_*Z*_*l*__^*R*^(*x*) < 1}, and then we can get *R*_*λ*_(*Z*_*l*_)⊆*R*_0.5_(*Z*_*l*_). To simplify the proof, let {*x*∣0.5 < *λ* ⩽ *μ*_*Z*_*l*__^*R*^(*x*) < 1} = [*x*_*i*_1__]_*R*_ ∪ [*x*_*i*_2__]_*R*_ ∪ ⋯∪[*x*_*i*_*q*__]_*R*_ and *q* ⩽ *k* in this paper. So, $Z_{l}\cap R_{\lambda }(Z_{l})=Z_{l}\cap (\underline{R}(Z_{l})\cup {\left[x_{i_{1}}\right]}_{R}\cup {\left[x_{i_{2}}\right]}_{R}\cup \cdots \cup {\left[x_{i_{q}}\right]}_{R})$. Because the intersection sets between any two elements in $\underline{R}(Z_{l}),{\left[x_{i_{1}}\right]}_{R},{\left[x_{i_{2}}\right]}_{R},\ldots ,{\left[x_{i_{k}}\right]}_{R}$ are empty sets, we can easily get that $|Z_{l}\cap R_{0.5}(Z_{l})|=|Z_{l}\cap \underline{R}(Z_{l})|+|Z_{l}\cap {\left[x_{i_{1}}\right]}_{R}|+|Z_{l}\cap {\left[x_{i_{2}}\right]}_{R}|+\cdots$ + $\left|Z_{l}\cap {\left[x_{i_{k}}\right]}_{R}|=|\underline{R}(Z_{l})|+|Z_{l}\cap {\left[x_{i_{1}}\right]}_{R}\right|$ + |*Z*_*l*_∩[*x*_*i*_2__]_*R*_ | +⋯+|*Z*_*l*_∩[*x*_*i*_*k*__]_*R*_|. And

(30) |Zl∩R0.5(Zl)||Zl∪R0.5(Zl)| =(|R_(Zl)|+|Zl∩[xi1]R|+⋯+|Zl∩[xiq]R|  +|Zl∩[xiq+1]R|+⋯+|Zl∩[xik]R|)  ×(|Zl|+|([xi1]R−Zl)|+⋯+|([xiq]R−Zl)|  +|([xiq+1]R−Zl)|+⋯+|([xik]R−Zl)|)−1,

and we have

(31) |Zl∩Rλ(Zl)||Zl∪Rλ(Zl)| =(|R_(Zl)|+|Zl∩[xi1]R|+|Zl∩[xi2]R|  +⋯+|Zl∩[xiq]R|)  ×(|Zl|+|([xi1]R−Zl)|+|([xi2]R−Zl)|  +⋯+|([xiq]R−Zl)|)−1.

And because |*Z*_*l*_ ∩ [*x*_*i*_*q*+1__]_*R*_ | +⋯+|*Z*_*l*_ ∩ [*x*_*i*_*k*__]_*R*_ | ⩾|([*x*_*i*_*q*+1__]_*R*_ − *Z*_*l*_)|+⋯ + |([*x*_*i*_*k*__]_*R*_ − *Z*_*l*_)|, according to Lemma 10, the inequality

(32) $$\begin{array}{c} \frac{\left|Z_{l}\cap R_{0.5}\left(Z_{l}\right)\right|}{\left|Z_{l}\cup R_{0.5}\left(Z_{l}\right)\right|}⩾\frac{\left|Z_{l}\cap R_{\lambda }\left(Z_{l}\right)\right|}{\left|Z_{l}\cup R_{\lambda }\left(Z_{l}\right)\right|}⩾\frac{\left|Z_{l}\cap \underline{R}\left(Z_{l}\right)\right|}{\left|Z_{l}\cup \underline{R}\left(Z_{l}\right)\right|} \end{array}$$

is held. Therefore, we have

(33) $$\begin{array}{c} \frac{\left|Z_{l}\cap R_{0.5}\left(Z_{l}\right)\right|}{2\left|Z_{l}\cup R_{0.5}\left(Z_{l}\right)\right|}⩾\frac{\left|Z_{l}\cap R_{\lambda }\left(Z_{l}\right)\right|}{2\left|Z_{l}\cup R_{\lambda }\left(Z_{l}\right)\right|}⩾\frac{\left|Z_{l}\cap \underline{R}\left(Z_{l}\right)\right|}{2\left|Z_{l}\cup \underline{R}\left(Z_{l}\right)\right|}. \end{array}$$

(2) The inequality

(34) $$\begin{array}{c} \frac{\left|Z_{u}\cap R_{0.5}\left(Z_{u}\right)\right|}{2\left|Z_{u}\cup R_{0.5}\left(Z_{u}\right)\right|}⩾\frac{\left|Z_{u}\cap R_{\lambda }\left(Z_{u}\right)\right|}{2\left|Z_{u}\cup R_{\lambda }\left(Z_{u}\right)\right|}⩾\frac{\left|Z_{u}\cap \underline{R}\left(Z_{u}\right)\right|}{2\left|Z_{u}\cup \underline{R}\left(Z_{u}\right)\right|} \end{array}$$

can be easily proved in a similar way to (1).

According to (1) and (2), the inequality $S(Z,R_{0.5}(Z))⩾S(Z,R_{\lambda }(Z))⩾S(Z,\underline{R}(Z))$ is held.

Based on Theorems 13 and 14, Corollary 15 can be obtained easily as follows.

**Corollary 15 .**

Let *U* be a finite domain, *Z* an interval set on *U*, and *R* an equivalence relation on *U*. *Z*⊆2^*U*^. If

(35) $$\begin{array}{c} \frac{\left|Z_{l}\right|}{\left|\bar{R}\left(Z_{l}\right)\right|}>\frac{\left|Z_{l}-R_{\lambda }\left(Z_{l}\right)\right|}{\left|\bar{R}\left(Z_{l}\right)-R_{\lambda }\left(Z_{l}\right)-Z_{l}\right|},\\ \frac{\left|Z_{u}\right|}{\left|\bar{R}\left(Z_{u}\right)\right|}>\frac{\left|Z_{u}-R_{\lambda }\left(Z_{u}\right)\right|}{\left|\bar{R}\left(Z_{u}\right)-R_{\lambda }\left(Z_{u}\right)-Z_{u}\right|}, \end{array}$$

then $S(Z,R_{0.5}(Z))⩾S(Z,R_{\lambda }(Z))⩾S(Z,\bar{R}(Z))$.

**Theorem 16 .**

Let *U* be a finite domain, *Z* an interval set on *U*, and *R* an equivalence relation on *U*. if 0.5 ⩽ *λ*_1_ < *λ*_2_ ⩽ 1, then *S*(*Z*, *R*_*λ*_1__(*Z*))⩾*S*(*Z*, *R*_*λ*_2__(*Z*)).

For example, *U*/*R* = {{*x*_1_}, {*x*_2_, *x*_3_}, {*x*_4_, *x*_5_, *x*_6_}, {*x*_7_, *x*_8_, *x*_9_, *x*_10_}}, *Z*_*u*_ = {*x*_1_, *x*_2_, *x*_3_, *x*_5_, *x*_6_, *x*_7_}, *Z*_*l*_ = {*x*_1_, *x*_2_, *x*_3_, *x*_5_, *x*_6_}. Then, $\bar{R}\left(Z_{u}\right)=\left\{x_{1},x_{2},x_{3},x_{4},x_{5},x_{6},x_{7},x_{8},x_{9},x_{10}\right\}$, $\underline{R}\left(Z_{u}\right)=\left\{x_{1},x_{2},x_{3}\right\}$, *R*_0.6_(*Z*_*u*_) = {*x*_1_, *x*_2_, *x*_3_, *x*_4_, *x*_5_, *x*_6_}, *R*_0.8_(*Z*_*u*_) = {*x*_1_, *x*_2_, *x*_3_}, $\bar{R}\left(Z_{l}\right)=\left\{x_{1},x_{2},x_{3},x_{4},x_{5},x_{6}\right\}$, $\underline{R}\left(Z_{l}\right)=\left\{x_{1},x_{2},x_{3}\right\}$, *R*_0.6_(*Z*_*l*_) = {*x*_1_, *x*_2_, *x*_3_, *x*_4_, *x*_5_, *x*_6_}, *R*_0.8_(*Z*_*l*_) = {*x*_1_, *x*_2_, *x*_3_}.

And then we can have *S*(*Z*, *R*_0.6_(*Z*)) = (5/(2 × 6)) + (5/(2 × 7)) = 65/84, *S*(*Z*, *R*_0.8_(*Z*)) = (3/(2 × 5)) + (3/(2 × 7)) = 18/35, *S*(*Z*, *R*_0.6_(*Z*)) > *S*(*Z*, *R*_0.8_(*Z*)). This example is in accordance with the theorem.

**Proof**

(1) For any *x* ∈ *R*_*λ*_1__(*Z*_*l*_), we have

(36) $$\begin{array}{c} \mu_{Z_{l}}^{R}\left(x\right)=\frac{\left|{\left[x\right]}_{R}\cap Z_{l}\right|}{\left|{\left[x\right]}_{R}\right|}⩾\lambda_{1}⩾0.5. \end{array}$$

Because *R* is an equivalence relation on *U*, all the classifications induced by *R* can be denoted by [*x*_1_]_*R*_, [*x*_2_]_*R*_,…, [*x*_*n*_]_*R*_. We have *R*_*λ*_1__(*Z*_*l*_) = {*x*∣*μ*_*Z*_*l*__^*R*^(*x*)⩾*λ*_1_} = {*x*∣*μ*_*Z*_*l*__^*R*^(*x*) = 1}∪{*x*∣*λ*_1_ ⩽ *μ*_*Z*_*l*__^*R*^(*x*) < 1} and $\left\{x|\mu_{Z_{l}}^{R}(x)=1\}=\underline{R}(Z_{l}\right)$ as well as {*x*∣*λ*_1_ ⩽ *μ*_*Z*_*l*__^*R*^(*x*) < 1} = [*x*_*i*_1__]_*R*_ ∪ [*x*_*i*_2__]_*R*_ ∪ ⋯∪[*x*_*i*_*u*__]_*R*_. We can also get *R*_*λ*_2__(*Z*_*l*_) = {*x*∣*μ*_*Z*_*l*__^*R*^(*x*)⩾*λ*_2_} = {*x*∣*μ*_*Z*_*l*__^*R*^(*x*) = 1}∪{*x*∣*λ*_2_ ⩽ *μ*_*Z*_*l*__^*R*^(*x*) < 1}. Let {*x*∣*λ*_2_ ⩽ *μ*_*Z*_*l*__^*R*^(*x*) < 1} = [*x*_*i*_1__]_*R*_ ∪ [*x*_*i*_2__]_*R*_ ∪ ⋯∪[*x*_*i*_*v*__]_*R*_, where 0.5 ⩽ *λ*_1_ < *λ*_2_ ⩽ 1 and *v* ⩽ *u*. So, $Z_{l}\cap R_{\lambda_{1}}(Z_{l})=Z_{l}\cap (\underline{R}(Z_{l})\cup {\left[x_{i_{1}}\right]}_{R}\cup {\left[x_{i_{2}}\right]}_{R}\cup \cdots \cup {\left[x_{i_{v}}\right]}_{R}\cup \cdots$ *∪*[*x*_*i*_*u*__]_*R*_), $Z_{l}\cap R_{\lambda_{2}}(Z_{l})=Z_{l}\cap (\underline{R}(Z_{l})\cup {\left[x_{i_{1}}\right]}_{R}\cup {\left[x_{i_{2}}\right]}_{R}\cup \cdots \cup {\left[x_{i_{v}}\right]}_{R})$. And because the intersection sets between any two elements in $\underline{R}(Z_{l})$, [*x*_*i*_1__]_*R*_, [*x*_*i*_2__]_*R*_,…, [*x*_*i*_*u*__]_*R*_ are empty sets, we have

(37) |Zl∩Rλ1(Zl)|=|Zl∩R_(Zl)|+|Zl∩[xi1]R|+|Zl∩[xi2]R|+⋯+|Zl∩[xiv]R|+⋯+|Zl∩[xiu]R|=|R_(Zl)|+|Zl∩[xi1]R|+|Zl∩[xi2]R|+⋯+|Zl∩[xiv]R|+⋯+|Zl∩[xiu]R|,|Zl∩Rλ2(Zl)|=|R_(Zl)|+|Zl∩[xi1]R|+|Zl∩[xi2]R|+⋯+|Zl∩[xiv]R|.

According to *Z*_*l*_ ∪ *R*_*λ*_1__(*Z*_*l*_) = *Z*_*l*_ ∪ ([*x*_*i*_1__]_*R*_ − *Z*_*l*_)*∪*([*x*_*i*_2__]_*R*_ − *Z*_*l*_)∪⋯∪([*x*_*i*_*v*__]_*R*_ − *Z*_*l*_)∪⋯∪([*x*_*i*_*u*__]_*R*_ − *Z*_*l*_) and because the intersection sets between any two elements in *Z*_*l*_, ([*x*_*i*_1__]_*R*_ − *Z*_*l*_), ([*x*_*i*_2__]_*R*_ − *Z*_*l*_),…, ([*x*_*i*_*v*__]_*R*_ − *Z*_*l*_),…, ([*x*_*i*_*u*__]_*R*_ − *Z*_*l*_) are empty sets, we easily have

(38) |Zl∪Rλ1(Zl)|=|Zl|+|([xi1]R−Zl)|+|([xi2]R−Zl)|+⋯+|([xiv]R−Zl)|+⋯+|([xiu]R−Zl)|,|Zl∪Rλ2(Zl)|=|Zl|+|([xi1]R−Zl)|+|([xi2]R−Zl)|+⋯+|([xiv]R−Zl)|.

Therefore,

(39) |Zl∩Rλ1(Zl)||Zl∪Rλ1(Zl)| =(|R_(Zl)|+|Zl∩[xi1]R|+⋯+|Zl∩[xiv]R|  +|Zl∩[xiv+1]R|+⋯+|Zl∩[xiu]R|)  ×(|Zl|+|([xi1]R−Zl)|+⋯+|([xiv]R−Zl)|  +|([xiv+1]R−Zl)|+⋯+|([xiu]R−Zl)|)−1,|Zl∩Rλ2(Zl)||Zl∪Rλ2(Zl)| =|R_(Zl)|+|Zl∩[xi1]R|+⋯+|Zl∩[xiv]R||Zl|+|([xi1]R−Zl)|+⋯+|([xiv]R−Zl)|.

According to

(40) μZlR(xiv+1)=|[xiv+1]R∩Zl||[xiv+1]R|=|[xiv+1]R∩Zl||[xiv+1]R∩Zl|+|[xiv+1]R−Zl|⩾λ1⩾0.5,

we have |[*x*_*i*_*v*+1__]_*R*_∩*Z*_*l*_ | ⩾|[*x*_*i*_*v*+1__]_*R*_ − *Z*_*l*_|. According to |[*x*_*i*_*v*+2__]_*R*_∩*Z*_*l*_ | ⩾|[*x*_*i*_*v*+2__]_*R*_ − *Z*_*l*_ | ,…, |[*x*_*i*_*u*__]_*R*_∩*Z*_*l*_ | ⩾|[*x*_*i*_*u*__]_*R*_ − *Z*_*l*_|, we have |*Z*_*l*_∩[*x*_*i*_*v*+1__]_*R*_| + ⋯+|*Z*_*l*_∩[*x*_*i*_*u*__]_*R*_ | ⩾|([*x*_*i*_*v*+1__]_*R*_ − *Z*_*l*_)|+⋯+|([*x*_*i*_*u*__]_*R*_ − *Z*_*l*_)|. And based on Lemma 10, we can easily have

(41) |Zl∩Rλ2(Zl)||Zl∪Rλ2(Zl)| =|R_(Zl)|+|Zl∩[xi1]R|+⋯+|Zl∩[xiv]R||Zl|+|([xi1]R−Zl)|+⋯+|([xiv]R−Zl)| ⩽(|R_(Zl)|+|Zl∩[xi1]R|+⋯+|Zl∩[xiv]R|  +|Zl∩[xiv+1]R|+⋯+|Zl∩[xiu]R|)  ×(|Zl|+|([xi1]R−Zl)|+⋯+|([xiv]R−Zl)|  +|([xiv+1]R−Zl)|+⋯+|([xiu]R−Zl)|)−1 =|Zl∩Rλ1(Zl)||Zl∪Rλ1(Zl)|.

Therefore,

(42) $$\begin{array}{c} \frac{\left|Z_{l}\cap R_{\lambda_{2}}\left(Z_{l}\right)\right|}{2\left|Z_{l}\cup R_{\lambda_{2}}\left(Z_{l}\right)\right|}⩽\frac{\left|Z_{l}\cap R_{\lambda_{1}}\left(Z_{l}\right)\right|}{2\left|Z_{l}\cup R_{\lambda_{1}}\left(Z_{l}\right)\right|}. \end{array}$$

(2) In the same way as  (1), the inequality

(43) $$\begin{array}{c} \frac{\left|Z_{u}\cap R_{\lambda_{2}}\left(Z_{u}\right)\right|}{2\left|Z_{u}\cup R_{\lambda_{2}}\left(Z_{u}\right)\right|}⩽\frac{\left|Z_{u}\cap R_{\lambda_{1}}\left(Z_{u}\right)\right|}{2\left|Z_{u}\cup R_{\lambda_{1}}\left(Z_{u}\right)\right|} \end{array}$$

is held.

According to (1) and (2), the inequality *S*(*Z*, *R*_*λ*_1__(*Z*))⩾*S*(*Z*, *R*_*λ*_2__(*Z*)) is held. So, the proof of Theorem 16 has been completed successfully.

Theorems 14 and 16 show that the similarity degree between an interval set *Z* and its approximation set *R*_*λ*_(*Z*) is a monotonically decreasing function with the parameter *λ*, and the similarity degree reaches its maximum value when *λ* = 0.5.

## 5. The Change Rules of Similarity in Different Knowledge Granularity Spaces

In different Pawlak's approximation spaces with different knowledge granularities, the change rules of the uncertainty of rough set are a key issue [21, 22]. Many researchers try to discover the change rules of uncertainty in rough set model [23, 24]. And we also find many change rules of uncertain concept in different knowledge spaces in our other papers [20]. In this paper, we continue to discuss the change rules of the similarity degree *S*(*Z*, *R*_0.5_(*Z*)) in Pawlak's approximation spaces with different knowledge granularities. In this paper, we focus on discussing how the similarity degree between *Z* and *R*_0.5_(*Z*) changes when the granules are divided into more subgranules in Pawlak's approximation space. In other words, it is an important issue concerning how *S*(*Z*, *R*_0.5_(*Z*)) changes with different knowledge granularities in Pawlak's approximation space.

Let [*x*_1_]_*R*_, [*x*_2_]_*R*_,…, [*x*_*n*_]_*R*_ be classifications of *U* under equivalence relation *R*. Let [*x*_1_]_*R*′_, [*x*_2_]_*R*′_,…, [*x*_*n*_]_*R*′_ be classifications of *U* under equivalence relation *R*′. If *R*′⊆*R*, then [*x*_*i*_]_*R*′_⊆[*x*_*i*_]_*R*_ (1 ≤ *i* ≤ *n*). And then, *U*/*R*′ is called a refinement of *U*/*R*, which is written as $U/R^{\prime }\underline{≺}\;U/R$. If ∃*x*_*j*_ ∈ *U*, then [*x*_*j*_]_*R*′_ ⊂ [*x*_*j*_]_*R*_. And then, *U*/*R*′ is called a strict refinement of *U*/*R*, which is written as *U*/*R*′≺*U*/*R*.

Next, we will analyze the relationship between *S*(*Z*, *R*_0.5_(*Z*)) and *S*(*Z*, *R*_0.5_′(*Z*)). Let *U*/*R*′≺*U*/*R*; in other words, for all *x* ∈ *U*,  [*x*]_*R*′_⊆[*x*]_*R*_ is always satisfied, and ∃*y* ∈ *U*,  [*y*]_*R*′_ ⊂ [*y*]_*R*_. And then, there must be two or more granules in *U*/*R*′ whose union is [*y*]_*R*_. To simplify the proof, we suppose that there is just only one granule which is divided into two subgranules, denoted by [*x*_*i*_*t*__^1^]_*R*′_ and [*x*_*i*_*t*__^2^]_*R*′_ in *U*/*R*′, and other granules keep unchanged.

There are 9 cases, and only 6 cases are possible.

**Theorem 17 .**

Let *U* be a finite domain, *Z* an interval set on *U*, and *R* and *R*′ two equivalence relations on *U*. Let [*x*_*i*_*t*__]_*R*_ be one granule which is divided into two subgranules marked as [*x*_*i*_*t*__^1^]_*R*′_ and [*x*_*i*_*t*__^2^]_*R*′_. If

(44) $$\begin{array}{c} S\left(Z_{l},R_{0.5}\left(Z_{l}\right)\right)⩾\frac{\left|\left[x_{i_{t}}^{2}\right]\cap Z_{l}\right|}{\left|\left[x_{i_{t}}^{2}\right]-Z_{l}\right|},\\ S\left(Z_{u},R_{0.5}\left(Z_{u}\right)\right)⩾\frac{\left|\left[x_{i_{t}}^{2}\right]\cap Z_{u}\right|}{\left|\left[x_{i_{t}}^{2}\right]-Z_{u}\right|}. \end{array}$$

Then, *S*(*Z*, *R*_0.5_(*Z*)) ⩽ *S*(*Z*, *R*_0.5_′(*Z*)).

**Proof**

There are 6 possible cases which will be discussed one by one as follows.

(1) [*x*_*i*_*t*__]_*R*_ is contained in both positive region of *Z*_*l*_ and positive region of *Z*_*u*_. In this case, obviously *S*(*Z*, *R*_0.5_(*Z*)) = *S*(*Z*, *R*_0.5_′(*Z*)) is held.
(2) [*x*_*i*_*t*__]_*R*_ is contained in both positive region of *Z*_*l*_ and negative region of *Z*_*u*_. In this case, obviously, *S*(*Z*, *R*_0.5_(*Z*)) = *S*(*Z*, *R*_0.5_′(*Z*)) is held.
(3) [*x*_*i*_*t*__]_*R*_ is contained in both negative region of *Z*_*l*_ and negative region of *Z*_*u*_. In this case, obviously, *S*(*Z*, *R*_0.5_(*Z*)) = *S*(*Z*, *R*_0.5_′(*Z*)) is held.
(4) [*x*_*i*_*t*__]_*R*_ is contained in both negative region of *Z*_*l*_ and boundary region of *Z*_*u*_. In this case,
  (45) $$\begin{array}{c} \frac{\left|R_{0.5}\left(Z_{l}\right)\cap Z_{l}\right|}{2\left|R_{0.5}\left(Z_{l}\right)\cup Z_{l}\right|}=\frac{\left|R_{0.5}^{\prime }\left(Z_{l}\right)\cap Z_{l}\right|}{2\left|R_{0.5}^{\prime }\left(Z_{l}\right)\cup Z_{l}\right|} \end{array}$$

is held obviously. Next, we discuss the relationship between

(46) $$\begin{array}{c} \frac{\left|R_{0.5}\left(Z_{u}\right)\cap Z_{u}\right|}{2\left|R_{0.5}\left(Z_{u}\right)\cup Z_{u}\right|},\\ \frac{\left|R_{0.5}^{\prime }\left(Z_{u}\right)\cap Z_{u}\right|}{2\left|R_{0.5}^{\prime }\left(Z_{u}\right)\cup Z_{u}\right|}. \end{array}$$

Let $R_{0.5}(Z_{u})=\underline{R}(Z_{u})\cup {\left[x_{i_{1}}\right]}_{R}\cup {\left[x_{i_{2}}\right]}_{R}\cup \cdots \cup {\left[x_{i_{k}}\right]}_{R}$. Let BN_*R*_(*Z*_*u*_) = [*x*_*i*_1__]_*R*_ ∪ [*x*_*i*_2__]_*R*_ ∪ ⋯∪[*x*_*i*_*m*__]_*R*_ where *m*⩾*k*. When [*x*_*i*_*t*__]_*R*_ is in boundary region of *Z*_*u*_, we should further discuss this situation. To simplify the proof, we suppose that there is just only one granule marked as [*x*_*i*_*t*__]_*R*_ in *U*/*R* which is divided into two subgranules marked as [*x*_*i*_*t*__^1^]_*R*′_ and [*x*_*i*_*t*__^2^]_*R*′_ in *U*/*R*′. And the other granules keep unchanged.

(a) If *k* < *t* ⩽ *m*, then [*x*_*i*_*t*__]_*R*_⊄*R*_0.5_(*Z*_*u*_).
(1) If [*x*_*i*_*t*__^1^]_*R*′_⊆*R*_0.5_′(*Z*_*u*_), [*x*_*i*_*t*__^2^]_*R*′_⊄*R*_0.5_′(*Z*_*u*_). From the proof of Theorem 12, we know
  (47) |Zu∩R0.5(Zu)||Zu∪R0.5(Zu)| =(|R_(Zu)|+|Zu∩[xi1]R|+|Zu∩[xi2]R|  +⋯+|Zu∩[xik]R|)  ×(|Zu|+|([xi1]R−Zu)|+|([xi2]R−Zu)|  +⋯+|([xik]R−Zu)|)−1.

Because [*x*_*i*_*t*__^1^]_*R*′_ ∪ [*x*_*i*_*t*__^2^]_*R*′_ = [*x*_*i*_*t*__]_*R*_, [*x*_*i*_*t*__^1^]_*R*′_⊆*R*_0.5_′(*Z*_*u*_), and [*x*_*i*_*t*__^2^]_*R*′_⊄*R*_0.5_′(*Z*_*u*_), we have

(48) |Zu∩R0.5′(Zu)||Zu∪R0.5′(Zu)| =(|R_′(Zu)|+|Zu∩[xi1]R′|+|Zu∩[xi2]R′|  +⋯+|Zu∩[xik]R′|+|Zu∩[xit1]R′|)  ×(|Zu|+|([xi1]R′−Zu)|+|([xi2]R′−Zu)|  +⋯+|([xik]R′−Zu)|+|([xit1]R′−Zu)|)−1 =(|R_(Zu)|+|Zu∩[xi1]R|+|Zu∩[xi2]R|  +⋯+|Zu∩[xik]R|+|Zu∩[xit1]R′|)  ×(|Zu|+|([xi1]R−Zu)|+|([xi2]R−Zu)|  +⋯+|([xik]R−Zu)|+|([xit1]R′−Zu)|)−1.

For [*x*_*i*_1__^1^]_*R*′_⊆*R*_0.5_′(*Z*_*u*_), |[*x*_*i*_*t*__^1^]_*R*′_∩*Z*_*u*_ | /(|[*x*_*i*_*t*__^1^]_*R*′_∩*Z*_*u*_ | +|[*x*_*i*_*t*__^1^]_*R*′_ − *Z*_*u*_|)⩾0.5, which means |[*x*_*i*_*t*__^1^]_*R*′_∩*Z*_*u*_ | ⩾|[*x*_*i*_*t*__^1^]_*R*′_ − *Z*_*u*_|. According to Lemma 10, we have

(49) $$\begin{array}{c} \frac{\left|R_{0.5}\left(Z_{u}\right)\cap Z_{u}\right|}{2\left|R_{0.5}\left(Z_{u}\right)\cup Z_{u}\right|}⩽\frac{\left|R_{0.5}^{\prime }\left(Z_{u}\right)\cap Z_{u}\right|}{2\left|R_{0.5}^{\prime }\left(Z_{u}\right)\cup Z_{u}\right|}. \end{array}$$

(2) If [*x*_*i*_*t*__^1^]_*R*′_⊄*R*_0.5_′(*Z*_*u*_),  [*x*_*i*_*t*__^2^]_*R*′_⊄*R*_0.5_′(*Z*_*u*_), then
  (50) $$\begin{array}{c} \frac{\left|R_{0.5}\left(Z_{u}\right)\cap Z_{u}\right|}{2\left|R_{0.5}\left(Z_{u}\right)\cup Z_{u}\right|}=\frac{\left|R_{0.5}^{\prime }\left(Z_{u}\right)\cap Z_{u}\right|}{2\left|R_{0.5}^{\prime }\left(Z_{u}\right)\cup Z_{u}\right|}. \end{array}$$

Because [*x*_*i*_*t*__]_*R*_⊄*R*_0.5_(*Z*_*u*_), the case that [*x*_*i*_1__^1^]_*R*′_⊆*R*_0.5_′(*Z*_*u*_) and [*x*_*i*_1__^2^]_*R*′_⊆*R*_0.5_′(*Z*_*u*_) is impossible.

(b) If 1 ⩽ *t* ⩽ *k*, then [*x*_*i*_*t*__]_*R*_⊆*R*_0.5_(*Z*_*u*_).
(1) If [*x*_*i*_*t*__^1^]_*R*′_⊆*R*_0.5_′(*Z*_*u*_) and [*x*_*i*_*t*__^2^]_*R*′_⊆*R*_0.5_′(*Z*_*u*_), then we can easily have
  (51) $$\begin{array}{c} \frac{\left|R_{0.5}\left(Z_{u}\right)\cap Z_{u}\right|}{2\left|R_{0.5}\left(Z_{u}\right)\cup Z_{u}\right|}=\frac{\left|R_{0.5}^{\prime }\left(Z_{u}\right)\cap Z_{u}\right|}{2\left|R_{0.5}^{\prime }\left(Z_{u}\right)\cup Z_{u}\right|}. \end{array}$$
(2) If [*x*_*i*_*t*__^1^]_*R*′_⊆*R*_0.5_′(*Z*_*u*_) and [*x*_*i*_*t*__^2^]_*R*′_⊄*R*_0.5_′(*Z*_*u*_), then
  (52) $$\begin{array}{c} \frac{\left|R_{0.5}\left(Z_{u}\right)\cap Z_{u}\right|}{\left|R_{0.5}\left(Z_{u}\right)\cup Z_{u}\right|}⩾\frac{\left|\left[x_{i_{t}}^{2}\right]\cap Z_{u}\right|}{\left|\left[x_{i_{t}}^{2}\right]-Z_{u}\right|}. \end{array}$$

Because [*x*_*i*_*t*__^2^]_*R*′_⊄*R*_0.5_′(*Z*_*u*_), we have the following.

(i) If [*x*_*i*_*t*__^2^]_*R*′_∩*Z*_*u*_ = *ϕ*, then we have |*Z*_*u*_∩[*x*_*i*_*t*__^1^]_*R*_ | = |*Z*_*u*_∩[*x*_*i*_*t*__]_*R*_| and |([*x*_*i*_*t*__^1^]_*R*′_ − *Z*_*u*_)|<|([*x*_*i*_*t*__]_*R*_ − *Z*_*u*_)|. Therefore,
  (53) |Zu∩R0.5′(Zu)||Zu∪R0.5′(Zu)| =(|R_′(Zu)|+|Zu∩[xi1]R′|+|Zu∩[xi2]R′|  +⋯+|Zu∩[xit1]R′|+⋯+|Zu∩[xik]R′|)  ×(|Zu|+|([xi1]R′−Zu)|+|([xi2]R′−Zu)|  +⋯+|([xit1]R′−Zu)|+⋯+|([xik]R′−Zu)|)−1 =(|R_(Zu)|+|Zu∩[xi1]R|+|Zu∩[xi2]R|  +⋯+|Zu∩[xit]R|+⋯+|Zu∩[xik]R|)  ×(|Zu|+|([xi1]R−Zu)|+|([xi2]R−Zu)|  +⋯+|([xit1]R′−Zu)|+⋯+|([xik]R−Zu)|)−1 >(|R_(Zu)|+|Zu∩[xi1]R|+|Zu∩[xi2]R|  +⋯+|Zu∩[xik]R|)  ×(|Zu|+|([xi1]R−Zu)|+|([xi2]R−Zu)|  +⋯+|([xik]R−Zu)|)−1 =|Zu∩R0.5(Zu)||Zu∪R0.5(Zu)|.
(ii) If [*x*_*i*_*t*__^1^]_*R*′_⊆*Z*_*u*_, then |[*x*_*i*_*t*__^1^]_*R*′_∩*Z*_*u*_ | = |[*x*_*i*_*t*__^1^]_*R*′_|. Therefore,
  (54) |Zu∩R0.5′(Zu)||Zu∪R0.5′(Zu)| =(|R_′(Zu)|+|Zu∩[xi1]R′|+|Zu∩[xi2]R′|  +⋯+|Zu∩[xit1]R′|+⋯+|Zu∩[xik]R′|)  ×(|Zu|+|([xi1]R′−Zu)|+|([xi2]R′−Zu)|  +⋯+|([xit1]R′−Zu)|+⋯+|([xik]R′−Zu)|)−1 =(|R_(Zu)|+|Zu∩[xi1]R|+|Zu∩[xi2]R|  +⋯+|[xit1]R′|+⋯+|Zu∩[xik]R|)  ×(|Zu|+|([xi1]R−Zu)|+|([xi2]R−Zu)|  +⋯+|[xit−1]R−Zu|  +|[xit+1]R−Zu|⋯+|([xik]R−Zu)|)−1 =|Zu∩R0.5(Zu)|−|[xit2]R′∩Zu||Zu∪R0.5(Zu)|−|[xit2]R′−Zu|.

Because

(55) $$\begin{array}{c} \frac{\left|Z_{u}\cap R_{0.5}\left(Z_{u}\right)\right|}{\left|Z_{u}\cup R_{0.5}\left(Z_{u}\right)\right|}⩾\frac{\left|\left[x_{i_{t}}^{2}\right]\cap Z_{u}\right|}{\left|\left[x_{i_{t}}^{2}\right]-Z_{u}\right|}, \end{array}$$

according to Lemma 11, we have

(56) $$\begin{array}{c} \frac{\left|R_{0.5}\left(Z_{u}\right)\cap Z_{u}\right|}{\left|R_{0.5}\left(Z_{u}\right)\cup Z_{u}\right|}⩽\frac{\left|R_{0.5}^{\prime }\left(Z_{u}\right)\cap Z_{u}\right|}{\left|R_{0.5}^{\prime }\left(Z_{u}\right)\cup Z_{u}\right|}. \end{array}$$

(iii) If [*x*_*i*_*t*__^1^]_*R*′_⊆BN_*R*′_(*Z*_*u*_) and [*x*_*i*_*t*__^2^]_*R*′_⊆BN_*R*′_(*Z*_*u*_), because [*x*_*i*_*t*__^1^]_*R*′_⊆*R*_0.5_′(*Z*_*u*_) and [*x*_*i*_*t*__^2^]_*R*′_⊄*R*_0.5_′(*Z*_*u*_), we have
  (57) |Zu∩R0.5′(Zu)||Zu∪R0.5′(Zu)| =(|R_′(Zu)|+|Zu∩[xi1]R′|+|Zu∩[xi2]R′|  +⋯+|Zu∩[xit1]R′|+⋯+|Zu∩[xik]R′|)  ×(|Zu|+|([xi1]R′−Zu)|+|([xi2]R′−Zu)|  +⋯+|([xit1]R′−Zu)|+⋯+|([xik]R′−Zu)|)−1 =(|R_(Zu)|+|Zu∩[xi1]R|+|Zu∩[xi2]R|  +⋯+|[xit1]R′|+⋯+|Zu∩[xik]R|)  ×(|Zu|+|([xi1]R−Zu)|+|([xi2]R−Zu)|  +⋯+|[xit1]R′−Zu|⋯+|([xik]R−Zu)|)−1 =|Zu∩R0.5(Zu)|−|[xit2]R′∩Zu||Zu∪R0.5(Zu)|−|[xit2]R′−Zu|.

Because

(58) $$\begin{array}{c} \frac{\left|Z_{u}\cap R_{0.5}\left(Z_{u}\right)\right|}{\left|Z_{u}\cup R_{0.5}\left(Z_{u}\right)\right|}⩾\frac{\left|\left[x_{i_{t}}^{2}\right]\cap Z_{u}\right|}{\left|\left[x_{i_{t}}^{2}\right]-Z_{u}\right|}, \end{array}$$

according to Lemma 11, we have

(59) $$\begin{array}{c} \frac{\left|R_{0.5}\left(Z_{u}\right)\cap Z_{u}\right|}{\left|R_{0.5}\left(Z_{u}\right)\cup Z_{u}\right|}⩽\frac{\left|R_{0.5}^{\prime }\left(Z_{u}\right)\cap Z_{u}\right|}{\left|R_{0.5}^{\prime }\left(Z_{u}\right)\cup Z_{u}\right|}. \end{array}$$

According to (a) and (b) above, we have *S*(*Z*, *R*_0.5_(*Z*)) ⩽ *S*(*Z*, *R*_0.5_′(*Z*)) when

(60) $$\begin{array}{c} \frac{\left|R_{0.5}\left(Z_{u}\right)\cap Z_{u}\right|}{\left|R_{0.5}\left(Z_{u}\right)\cup Z_{u}\right|}⩾\frac{\left|\left[x_{i_{t}}^{2}\right]\cap Z_{u}\right|}{\left|\left[x_{i_{t}}^{2}\right]-Z_{u}\right|}. \end{array}$$

(5) [*x*_*i*_*t*__]_*R*_ is contained in boundary region of *Z*_*l*_ and positive region of *Z*_*u*_. In this case,
  (61) $$\begin{array}{c} \frac{\left|R_{0.5}\left(Z_{u}\right)\cap Z_{u}\right|}{\left|R_{0.5}\left(Z_{u}\right)\cup Z_{u}\right|}=\frac{\left|R_{0.5}^{\prime }\left(Z_{u}\right)\cap Z_{u}\right|}{\left|R_{0.5}^{\prime }\left(Z_{u}\right)\cup Z_{u}\right|}. \end{array}$$

Next, we discuss the relationship between

(62) $$\begin{array}{c} \frac{\left|R_{0.5}\left(Z_{l}\right)\cap Z_{l}\right|}{\left|R_{0.5}\left(Z_{l}\right)\cup Z_{l}\right|},\\ \frac{\left|R_{0.5}^{\prime }\left(Z_{l}\right)\cap Z_{l}\right|}{\left|R_{0.5}^{\prime }\left(Z_{l}\right)\cup Z_{l}\right|}. \end{array}$$

Similar to (a) and (b) in (4), when

(63) $$\begin{array}{c} \frac{\left|Z_{l}\cap R_{0.5}\left(Z_{l}\right)\right|}{\left|Z_{l}\cup R_{0.5}\left(Z_{l}\right)\right|}⩾\frac{\left|\left[x_{i_{t}}^{2}\right]\cap Z_{l}\right|}{\left|\left[x_{i_{t}}^{2}\right]-Z_{l}\right|}, \end{array}$$

we can get

(64) $$\begin{array}{c} \frac{\left|Z_{l}\cap R_{0.5}\left(Z_{l}\right)\right|}{\left|Z_{l}\cup R_{0.5}\left(Z_{l}\right)\right|}⩽\frac{\left|Z_{l}\cap R_{0.5}^{\prime }\left(Z_{l}\right)\right|}{\left|Z_{l}\cup R_{0.5}^{\prime }\left(Z_{l}\right)\right|}. \end{array}$$

So, in the condition, we can draw a conclusion that *S*(*Z*, *R*_0.5_(*Z*)) ⩽ *S*(*Z*, *R*_0.5_′(*Z*)).

(6) [*x*_*i*_*t*__]_*R*_ is contained in boundary region of *Z*_*l*_ and boundary region of *Z*_*u*_.

According to the proofs of (4) and (5), if  *S*(*Z*_*l*_, *R*_0.5_(*Z*_*l*_))⩾|[*x*_*i*_*t*__^2^]∩*Z*_*l*_|/|[*x*_*i*_*t*__^2^] − *Z*_*l*_| and *S*(*Z*_*u*_, *R*_0.5_(*Z*_*u*_))⩾|[*x*_*i*_*t*__^2^]∩*Z*_*u*_|/|[*x*_*i*_*t*__^2^] − *Z*_*u*_|, we easily have *S*(*Z*, *R*_0.5_(*Z*)) ⩽ *S*(*Z*, *R*_0.5_′(*Z*)).

From (1), (2), (3), (4), (5), and (6), Theorem 17 is proved successfully.

Theorem 17 shows that, under some conditions, the similarity degree between an interval set *Z* and its approximation set *R*_0.5_(*Z*) is a monotonically increasing function when the knowledge granules in *U*/*R* are divided into many finer subgranules in *U*/*R*′, where *U*/*R*′ is a refinement of *U*/*R*.

## 6. Conclusion

With the development of uncertain artificial intelligence, the interval set theory attracts more and more researchers and gradually develops into a complete theory system. The interval set theory has been successfully applied to many fields, such as machine learning, knowledge acquisition, decision-making analysis, expert system, decision support system, inductive inference, conflict resolution, pattern recognition, fuzzy control, and medical diagnostics systems. It is an important tool of granular computing as well as the rough set which is one of the three main tools of granular computing [25, 26]. In the interval set theory, the target concept is approximately described by two certain sets, that is, the upper bound and lower bound. In other words, the essence of this theory is that we deal with the uncertain problems with crisp set theory method. Many researches have been completed on extended models of the interval set, but the theories nearly cannot present better approximation set of the interval set *Z*. In this paper, the approximation set *R*_0.5_(*Z*) of target concept *Z* in current knowledge space is proposed from a new viewpoint and related properties are analyzed in detail.

In this paper, the interval set is transformed into a fuzzy set at first, and then the uncertain elements in boundary region are classified by cut-set with some threshold. Next, the approximation set *R*_0.5_(*Z*) of the interval set *Z* is defined and the change rules of *S*(*Z*, *R*_0.5_(*Z*)) in different knowledge granularity spaces are analyzed. These researches show that *R*_0.5_(*Z*) is a better approximation set of *Z* than both $\bar{R}(Z)$ and $\underline{R}(Z)$. Finally, a kind of crisp approximation set of interval set is proposed in this paper. These researches present a new method to describe uncertain concept from a special viewpoint, and we hope these results can promote the development of both uncertain artificial intelligence and granular computing and extend the interval set model into more application fields. It is an important research issue concerning discovering more knowledge and rules from the uncertain information [27]. The fuzzy set and the rough set have been used widely [28–32]. Recently, the interval set theory is applied to many important fields, such as software testing [33], the case generation based on interval combination [34], and incomplete information table [35–38]. In the future research, we will focus on acquiring the approximation rules from uncertain information systems based on the approximation sets of an interval set.
